# Impact of gender differences on prognosis in patients with acute cerebral infarction associated with REM-related obstructive sleep apnea: a retrospective cohort study

**DOI:** 10.3389/fneur.2026.1723917

**Published:** 2026-02-06

**Authors:** Yan Liu, Yuxin Ye, Liwen Xu, Shutong Sun, Tianyu Jing, Gang Xu, Tieyu Tang, Cheng Chu

**Affiliations:** 1 Department of Neurology, The Affiliated Hospital of Yangzhou University, Yangzhou University, Yangzhou, China; 2 Center for Sleep Medicine, The Affiliated Hospital of Yangzhou University, Yangzhou, China; 3 Faculty of Medicine, School of Nursing, Yangzhou University, Yangzhou, China; 4 The First School of Clinical Medicine, Faculty of Medicine, Yangzhou University, Yangzhou, China

**Keywords:** acute cerebral infarction (ACI), gender differences, prognosis, REM-related obstructive sleep apnea (REM-OSA), retrospective cohort study

## Abstract

**Background:**

Acute cerebral infarction (ACI) comorbid with rapid eye movement-related obstructive sleep apnea (REM-OSA) worsens prognosis, but gender-specific differences in outcomes and their underlying mechanisms remain understudied, and this gap limits the development of personalized interventions for female patients.

**Objective:**

To analyze gender differences in prognosis among ACI patients with REM-OSA and identify contributing factors, providing evidence for improving outcomes.

**Methods:**

A retrospective analysis of acute cerebral infarction (ACI) patients with REM-related obstructive sleep apnea (REM-OSA) admitted to the Department of Neurology, Affiliated Hospital of Yangzhou University, from February 2023 to February 2025. The study included 140 patients (71 females, 69 males) with ACI and REM-OSA, who underwent all-night polysomnographic monitoring. The data collected included: (1) baseline clinical characteristics; (2) laboratory indicators; (3) subjective sleep assessments, including sleep quality, daytime sleepiness, and emotional state; (4) objective sleep monitoring data (video polysomnography during the night); (5) prognostic indicators, including the Modified Rankin Scale (mRS) and the National Institutes of Health Stroke Scale (NIHSS).

**Results:**

Female patients had significantly higher discharge NIHSS scores (*p* = 0.011) and 3-month mRS scores (*p* = 0.008). Multivariate regression analysis showed that the lowest nadir of oxyhemoglobin saturation (SpO₂) (*β* = −0.325, *p* = 0.006) and neutrophil count (*β* = 0.258, *p* = 0.019) were independent prognostic predictors for poor outcomes in female patients.

**Conclusion:**

Female ACI patients with REM-OSA have poorer prognosis, likely linked to severe REM hypoxia and inflammation. Gender-specific REM-OSA screening and interventions are warranted.

## 1 Introduction

Acute cerebral infarction (ACI) is a common and serious cerebrovascular disease, characterized by high morbidity, high disability rate, and high mortality, which imposes a heavy burden on patients and their families. Previous studies have shown that the global incidence of ACI is on the rise and has become an important factor threatening human health and quality of life (1, 2). Its core pathogenesis is the obstruction of cerebral blood vessels by thrombi or emboli, leading to ischemic and hypoxic necrosis of brain tissue, which in turn causes a series of neurological deficit symptoms such as disturbance of consciousness, language and motor dysfunction, and sensory abnormalities (3).

Obstructive sleep apnea (OSA) is a sleep-related breathing disorder characterized by partial or complete collapse of the upper airway during sleep, leading to disrupted sleep structure and intermittent hypoxemia. OSA is commonly associated with various chronic diseases and has become one of the significant public health concerns in current society (4). Rapid eye movement–related obstructive sleep apnea (REM-OSA), an important clinical subtype of OSA, specifically refers to apnea or hypopnea events primarily occurring during rapid eye movement (REM) sleep. REM-OSA can lead to more severe and longer-lasting hypoxemia, accompanied by increased sympathetic nervous activity (5).

The comorbidity of REM-OSA in patients with ACI is a common clinical phenomenon. Studies have shown that compared with cerebral infarction patients with non-rapid eye movement-related obstructive sleep apnea (NREM-OSA), those with REM-OSA have worse neurological recovery and prognosis (6). The intermittent hypoxia (IH) state in OSA patients can cause neuronal damage (especially involving the hippocampus and cortex), which in turn leads to cognitive dysfunction, a serious and specific complication in OSA patients (7). The mechanisms affecting the prognosis of acute ACI patients with REM-OSA are multifactorial. After the occurrence of cerebral infarction, the supply of oxygenated blood is insufficient, and neurons in the affected brain regions are rapidly lost (3), while the physiological characteristics of the REM sleep stage itself (such as reduced upper airway muscle tone) will further aggravate respiratory disorders, forming a vicious circle (4). In addition, the abnormal expression of inflammatory factors (such as elevated neutrophil count) may promote blood–brain barrier disruption, cerebral edema, and brain injury, thereby further affecting the prognosis (8).

Previous studies have shown that female stroke patients differ significantly from male patients in terms of risk factor profiles, ischemic stroke subtype distribution, stroke severity, and clinical outcomes. These sex-related differences may further influence functional recovery and long-term prognosis after stroke. Currently, there is still relatively limited research on ACI patients with comorbid REM-OSA, particularly regarding the differences in prognosis and influencing factors between male and female patients. Sex differences play an important role in the onset, progression, and prognosis of various diseases, and this phenomenon may also exist in ACI patients with comorbid REM-OSA. Therefore, it is of significant clinical and practical value to investigate the differences in prognosis and the related influencing factors between male and female ACI patients with REM-OSA.

This study is a retrospective analysis based on hospital medical records, and the data were sourced from the standardized clinical database established by the Department of Neurology at the Affiliated Hospital of Yangzhou University. This database systematically integrates baseline information, diagnostic and treatment records, laboratory test results, and special examination findings of hospitalized patients, providing comprehensive data support for the study. However, since this is a retrospective study and polysomnography was not routinely performed in all acute cerebral infarction patients, there may be selection bias in the study population. Patients who underwent polysomnography were typically those with more prominent sleep-related symptoms and relatively preserved neurological function, which may somewhat limit the generalizability of the study results.

## 2 Materials and methods

### 2.1 Participants

The study participants were acute ischemic stroke patients who were treated in the Department of Neurology at the Affiliated Hospital of Yangzhou University from February 2024 to May 2025, and who underwent overnight polysomnography (PSG) according to the standards of the American Academy of Sleep Medicine (AASM) during their hospitalization. The diagnosis of acute ischemic stroke strictly followed the relevant criteria in the Chinese Guidelines for the Diagnosis and Treatment of Acute Ischemic Stroke (2018) (9) and was confirmed by head computed tomography (CT) or magnetic resonance imaging (MRI). The specific diagnostic criteria are as follows: (1) a clear history of acute ischemic stroke onset; (2) focal neurological deficits found during neurological physical examination; (3) acute ischemic lesions identified by head MRI combined with diffusion-weighted imaging (DWI), or acute ischemic lesions confirmed by head CT after exclusion of intracerebral hemorrhage. Based on previous studies and commonly used clinical diagnostic standards, REM-OSA is defined as a REM apnea-hypopnea index (REM-AHI) ≥ 5 events/h and a non-REM apnea-hypopnea index (NREM-AHI) < 5 events/h, with total REM sleep duration ≥30 min (10).

This threshold has been used in multiple studies to define REM-related obstructive sleep apnea, particularly in populations with few respiratory events during non-REM sleep but significant breathing disturbances during REM sleep. Although some studies have proposed using a higher REM-AHI cutoff to identify individuals at higher cardiovascular risk, this study adopts a REM-AHI ≥ 5 events/h as the diagnostic standard, aiming to improve the sensitivity of identifying REM-related breathing abnormalities, particularly in acute ischemic stroke patients.

### 2.2 Inclusion and exclusion criteria

This retrospective cohort study included patients diagnosed with cerebral infarction at the Department of Neurology, Affiliated Hospital of Yangzhou University, from February 2023 to February 2025. Clinical data and both subjective and objective sleep data from the sleep center were collected. Three months after discharge, follow-up was completed, and neurological function scores were collected to assess the primary outcomes. The inclusion criteria were as follows: (1) age ≥18 years; (2) diagnosis of ischemic stroke according to the Chinese Guidelines for the Diagnosis and Treatment of Acute Ischemic Stroke (2018) (9), confirmed by head CT or MRI; (3) completion of overnight polysomnography (PSG) within 24–48 h after admission, when the patient was in a relatively stable condition. The sleep data obtained met technical quality standards for interpretation, including EEG, EOG, chin EMG, airflow, chest-abdominal movements, and oxygen saturation signals, and were sufficient for sleep staging and respiratory event analysis based on standards. The exclusion criteria were as follows: (1) known comorbidity of OSA with prior positive pressure ventilation therapy; (2) PSG showing a total REM sleep duration of < 30 min; (3) history of oropharyngeal reconstructive surgery.

This study has been approved by the Ethics Committee of Yangzhou University (Ethics Approval No. 2023-YKL09), and all patients signed informed consent forms before enrollment.

### 2.3 Demographic and clinical data

The baseline data included in this study analysis comprised age, gender, body mass index (BMI), smoking history, drinking history, and past medical history. The blood-related test indicators included white blood cell count, neutrophil count, triglyceride level, total cholesterol level, serum creatinine level, and glycated hemoglobin level.

### 2.4 Questionnaire assessment and outcome measurement

The Epworth sleepiness scale (ESS) is the most commonly used scale for assessing the degree of daytime sleepiness in patients with sleep disorders (11). This scale consists of 8 items, which is used to assess the subject’s tendency to sleepiness in 8 different scenarios over the past month. The total score is 24 points: a score >6 indicates the presence of sleepiness, a score >11 indicates excessive sleepiness, and a score >16 indicates severe sleepiness.

The Pittsburgh sleep quality index (PSQI) is used to assess the sleep quality of subjects and has good reliability and validity in the assessment of sleep quality among patients with mental illnesses, sleep disorders, and various physical diseases (12). This scale consists of 9 items, and its scoring system covers 7 dimensions: sleep quality, sleep latency, sleep duration, sleep efficiency, sleep disturbances, use of sleep medications, and daytime dysfunction. The total score ranges from 0 to 21 points, with higher scores indicating poorer sleep quality. Given that the PSQI is a non-specific subjective sleep quality measure, its results may be influenced by various factors, such as neurological function status, mood, and physical discomfort. Therefore, in this study, PSQI was used only for descriptive analysis of overall sleep quality and was not used as the basis for specific conclusions related to REM-OSA.

The Hospital anxiety and depression scale (HADS) is a commonly used scale in clinical practice for assessing the emotional states (anxiety and depression) and their severity in non-psychiatric patients (13). This scale consists of 14 items, divided into an anxiety subscale (HADS-A, 7 items) and a depression subscale (HADS-D, 7 items). The HADS-A is used to assess anxiety-related symptoms (such as tension, worry, and a sense of panic) in subjects over the past month, while the HADS-D is used to assess depression-related symptoms (such as low mood, diminished interest, and lack of energy) over the same period. Each item of the scale is scored on a 0–3 scale, and each of the two subscales has a total score of 21 points: scores of 0–7 indicate no obvious anxiety/depression, scores of 8–10 indicate mild anxiety/depression, scores of 11–14 indicate moderate anxiety/depression, and scores of 15–21 indicate severe anxiety/depression. In this study, the HADS was included to assess psychological factors such as anxiety and depression, which may impact subjective sleep perceptions, sleep structure, and post-stroke neurological recovery. Therefore, HADS was primarily used as an assessment tool for potential confounding factors and was adjusted for in the statistical analysis, rather than being used as a primary outcome measure.

### 2.5 Neurologic function assessment

The National Institutes of Health Stroke Scale (NIHSS) is a comprehensive and objective semiquantitative assessment tool for stroke severity, and it has prognostic value (14). It consists of a total of 42 points, with higher scores indicating more severe neurological damage. In this study, the NIHSS was used as a prognostic indicator to evaluate the severity of stroke in patients upon admission and discharge.

The Barthel index (BI) is used to assess the degree of disability in patients upon admission and their functional improvement after rehabilitation (15). This scale includes four grades, with a total score of 100 points; higher scores indicate greater independence and less dependence in patients. It has concise items, convenient operation, and high sensitivity, and has shown good reliability and validity in both face-to-face and telephone assessments.

The Modified Rankin Scale (mRS) is used to assess the neurological function recovery of stroke patients (16), with higher scores indicating poorer neurological function recovery. It was assessed 3 months after the patient’s discharge during the follow-up to reflect their short-term functional outcome.

The NIHSS and BI scores were assessed at the time of discharge to reflect neurological deficits and activities of daily living levels during the acute phase and at discharge. The mRS score was assessed at the 3-month follow-up after discharge to reflect short-term functional recovery outcomes. These three scales comprehensively evaluate the prognosis of stroke patients from different time points and functional perspectives.

### 2.6 Polysomnography

#### 2.6.1 Sleep monitoring

All patients underwent overnight video-polysomnography (vPSG) at the Sleep Monitoring Center of the Affiliated Hospital of Yangzhou University. The PSG monitoring parameters included: (1) Electroencephalography (EEG): EEG electrodes were placed using the international 10–20 system. The EEG channels used in this study included F3–A2, F4–A1, C3–A2, C4–A1, O1–A2, and O2–A1, to record electrical activity from different brain regions, supporting sleep staging and wakefulness scoring. (2) Electrooculography (EOG): Bilateral eye movement leads were used to monitor eye movements during sleep. (3) Electromyography (EMG): This included mentalis muscle EMG to assess changes in muscle tone during sleep, and bilateral anterior tibialis muscle EMG to assess limb movements and periodic limb movements. (4) Electrocardiography (ECG): Standard ECG monitoring was used to assess heart rhythm. (5) Respiratory Monitoring: Respiratory airflow was monitored using a nasal pressure sensor (nasal pressure cannula) combined with an oronasal thermistor. The nasal pressure sensor quantified nasal airflow changes, while the oronasal thermistor detected the presence and direction of airflow at the mouth and nose, enhancing the accuracy of respiratory event detection. (6) Respiratory Effort: Monitored using respiratory effort bands placed around the chest and abdomen to assess chest and abdominal movements. (7) Hypoventilation Monitoring: Ventilation status was assessed via transcutaneous carbon dioxide (tcCO₂) monitoring (17). tcCO₂ was measured continuously and non-invasively by slightly heating the sensor to arterialize local blood, with CO₂ pressure measurements being well correlated with arterial blood gas analysis. In this study, hypoventilation was defined as tcCO₂ levels sustained above 45 mmHg. The longest duration of hypoventilation was defined as the longest continuous period during which tcCO₂ exceeded this threshold, measured in minutes. (8) Oxygen Saturation: Continuously monitored using a pulse oximeter to assess oxygen levels during sleep. (9) Body Position Monitoring: Synchronous video recording was used to assist in event scoring and to identify body movements or artifacts that could affect the scoring.

All PSG data were independently scored by two trained sleep technicians according to The AASM Manual for the Scoring of Sleep and Associated Events: Rules, Terminology and Technical Specifications, (Version 3, 2023) (18). In case of disagreement, a third physician made the final determination. The analysis parameters in this study included REM latency, REM-AHI, oxygen desaturation index (ODI), defined as the number of events during sleep where oxygen saturation decreased by ≥3% from baseline (a threshold recommended by the AASM manual and widely used in polysomnography research), as well as the arousal index and respiratory-related arousal index, etc.

#### 2.6.2 Diagnostic and grouping criteria

Participants in this study were divided into male and female subgroups based on patient gender. The randomization principle was strictly followed during the grouping process to ensure comparability between the two subgroups in terms of baseline characteristics such as age and disease severity. Statistical analysis of baseline data [including age, body mass index (BMI), and past medical history] of patients in the two subgroups was conducted to verify group balance, aiming to reduce the interference of confounding factors on the study results.

#### 2.6.3 Statistical methods

An Excel database was created for this study, and data analysis was performed using the SPSS 27.0 software package (IBM Corp., Armonk, USA). Measurement data with a normal distribution were expressed as mean ± standard deviation ( $\bar{x}\pm s$ ), while measurement data with a non-normal distribution were expressed as median (quartile) [M (P25, P75)]; independent samples *t*-test was used for inter-group comparisons (applicable to normally distributed data). Count data were expressed as [n (%)], and chi-square (*χ*^2^) test was used for inter-group comparisons. Spearman rank correlation analysis was employed to explore the correlation between REM-AHI and other variables; linear regression models were used to analyse the association between 3-month mRS scores and REM-AHI, neutrophils, and minimum blood oxygen saturation, so as to clarify the impact of the aforementioned variables on 3-month mRS scores. Meanwhile, linear regression models were also used to analyse the association between REM-OSA-related indicators and prognostic indicators of cerebral infarction. All statistical tests were two-sided, and 95% confidence intervals (95% CI) were reported. Differences were considered statistically significant at (*p* < 0.05).

## 3 Results

### 3.1 Baseline demographic and clinical characteristics

A total of 140 patients with ACI and REM-OSA were included in this study, all of whom underwent overnight polysomnography. Among these, 69 were male patients, with an average age of 58.51 ± 13.53 years, and 71 were female patients, with an average age of 60.83 ± 12.45 years. The original patient cohort consisted of 168 participants, from which 28 patients with a global apnea-hypopnea index (AHI, calculated based on total sleep time) of less than 5 events/h were excluded. REM-AHI was used only for subsequent REM-OSA-related analysis.

Baseline data comparisons between the two groups are shown in Table 1. There was a significant difference in smoking rate between the two groups (*p* = 0.006), with a significantly higher smoking rate in male patients compared to female patients. Except for smoking status, there were no statistically significant differences between the groups in terms of education level, living arrangements, residence, employment status, work type, health insurance type, monthly income, medication use, alcohol consumption, hypertension history, diabetes history, and body mass index.

**Table 1:** Baseline demographic and clinical characteristics of the study population.

Variables	Female (*N* = 71)	Male (*N* = 69)	*χ*^2^/*t*	*p*
Educational level			3.757	0.289
Junior high school and below	48 (67.61)	43 (62.32)		
Senior high school	13 (18.31)	16 (23.19)		
Junior college	3 (4.23)	7 (10.14)		
Bachelor’s degree and above	7 (9.86)	3 (4.35)		
Living arrangement			3.847	0.279
Living alone	11 (15.49)	5 (7.25)		
Spouse	39 (54.93)	48 (69.57)		
Children	7 (9.86)	5 (7.25)		
Spouse and children	14 (19.72)	11 (15.94)		
Place of residence			0.015	0.903
Rural area	24(33.80)	23(33.33)		
Urban area	24(33.80)	45(65.22)		
Employment status			4.737	0.094
Employed	12(16.90)	19(27.54)		
Retired	36(50.70)	23(33.33)		
Not in the labor force	23(32.39)	27(39.13)		
Nature of work			0.048	0.826
Mental work	27 (38.03)	25 (36.23)		
Physical work	44 (61.97)	44 (63.77)		
Health insurance	64 (90.14)	62 (89.86)	0.003	0.955
Monthly income [RMB]			0.092	0.955
<5,000	51 (71.83)	50 (72.46)		
5,000–10,000	15 (21.13)	15 (21.74)		
>10,000	5 (7.04)	4 (5.80)		
Medication use			5.832	0.120
None	22 (30.99)	23 (33.33)		
1 kind	9 (12.68)	13 (18.84)		
2–3 kinds	22 (30.99)	10 (14.49)		
>3 kinds	18 (25.35)	23 (33.33)		
Smoking history [*n*, %]	13 (18.31)	27 (39.13)	7.433	0.006
Alcohol drinking history [*n*, %]	30 (42.25)	31 (44.93)	0.102	0.750
Hypertension history [*n*, %]	38 (53.52)	38 (55.07)	0.034	0.854
Diabetes mellitus history [*n*, %]	18 (25.35)	21 (30.43)	0.450	0.502
Age [y, ( $\bar{x}\pm s$ )]	60.85 ± 12.46	58.51 ± 13.53	1.064	0.289
BMI [kg/m^2^, ( $\bar{x}\pm s$ )]	26.05 ± 4.15	25.24 ± 3.44	1.264	0.208

BMI, body mass index.

**Figure 1:**
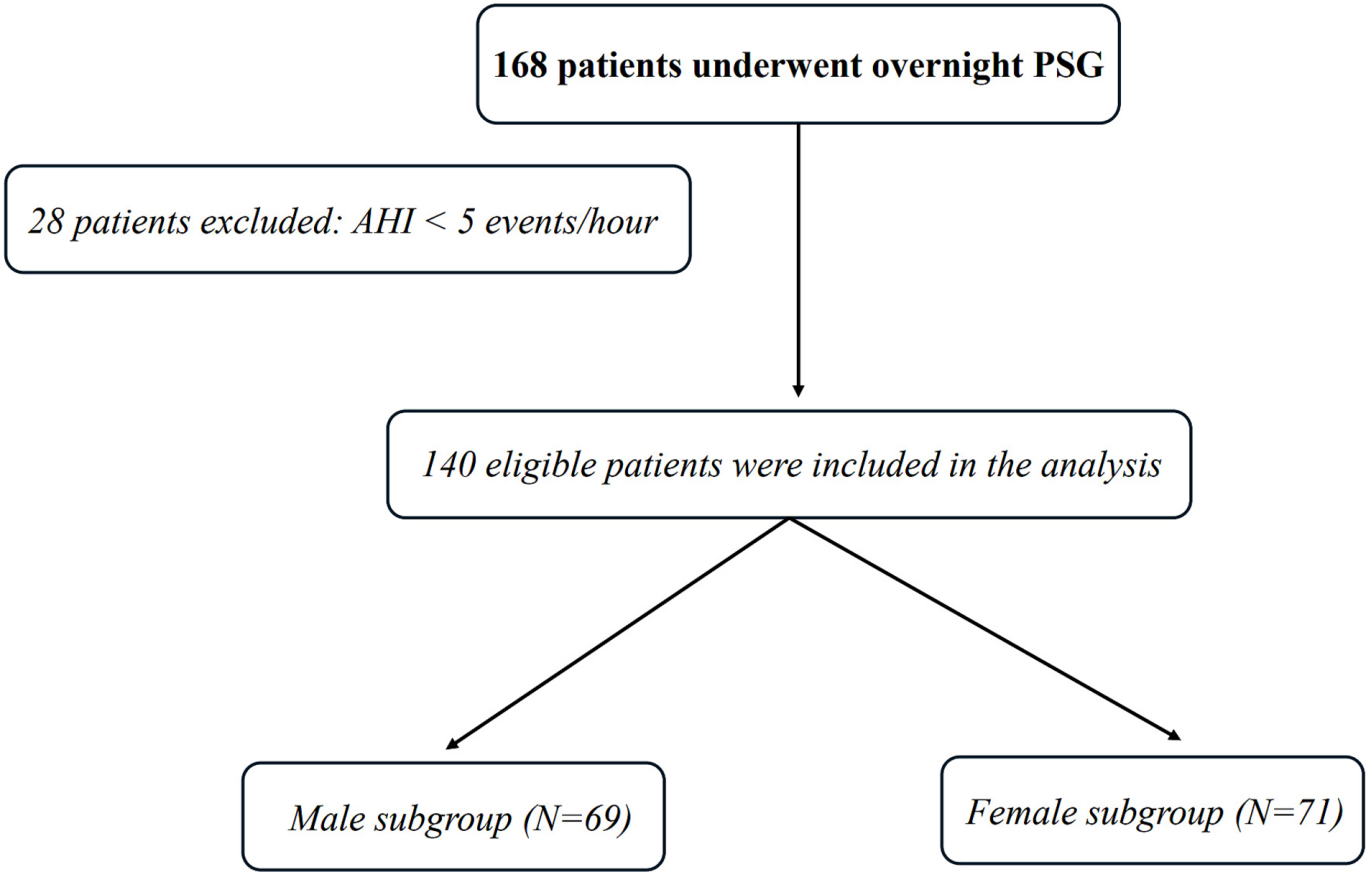
Consort flow chart of the study sample. PSG, polysomnography.

Given that smoking is an important known risk factor for OSA and a potential confounder, this study paid particular attention to its influence in the interpretation of results, in order to minimize its potential impact on the analysis of gender differences (see Figure 1).

### 3.2 Differences in laboratory indicators

The laboratory indicator differences between the two groups are shown in Table 2. There were significant differences in white blood cell count (*p* = 0.002) and neutrophil count (*p* = 0.011) between the two groups, while no significant differences were observed in triglycerides, total cholesterol, serum creatinine, or glycosylated hemoglobin levels. Further analysis revealed that female patients had significantly higher white blood cell and neutrophil counts compared to male patients. This difference may be related to gender-specific inflammatory responses or immune modulation characteristics, which requires further investigation for confirmation.

**Table 2:** Differences in laboratory indicators of study participants.

Variables	Female (*N* = 71)	Male (*N* = 69)	*t*	*p*
TG, mmol/L	1.56 ± 0.65	1.80 ± 1.52	−1.184	0.239
TC, mmol/L	4.39 ± 0.99	4.22 ± 0.97	1.075	0.284
SCR, μmol/L	72.29 ± 26.00	75.06 ± 19.84	−0.708	0.480
HbA1c, %	6.71 ± 2.21	6.69 ± 2.58	0.064	0.949
NE, 10^9^/L	7.06 ± 3.26	5.92 ± 1.75	2.585	0.011
WBC, 10^9^/L	10.03 ± 10.02	6.04 ± 2.11	3.179	0.002

TG, triglyceride; TC, total cholesterol; SCR, serum creatinine; HbA1c, glycosylated haemoglobin; WBC, white blood cell; NE, neutrophilic granulocyte.

### 3.3 Differences in sleep data

The polysomnography data of the patients are shown in Table 3. The results indicated that there was no statistically significant difference between the two groups in terms of the overall apnea-hypopnea index (total AHI). However, significant differences were observed in REM-related respiratory events and oxygenation indicators, particularly in sleep efficiency, REM-AHI, oxygen desaturation index, longest apneic time, longest hypoventilation time, minimum oxygen saturation, and respiratory-related arousal index. In contrast, no statistically significant differences were found between the two groups in total sleep time, Wake Time After Sleep Onset, sleep stage proportions (N1, N2, N3, and REM), sleep latency, REM latency, and arousal index.

**Table 3:** Comparison of polysomnography data of study participants.

Variables	Female (*N* = 71)	Male (*N* = 69)	*t*	*p*
Sleep time, min	443.77 ± 74.28	442.07 ± 67.96	0.141	0.888
Sleep efficiency	80.76 ± 9.80	84.55 ± 10.59	−2.199	0.030
WASO, min	77.68 ± 44.00	75.92 ± 67.77	0.183	0.855
N1 stage duration [min]	39.09 ± 41.15	38.93 ± 34.77	−0.024	0.565
N2 stage duration [min]	153.26 ± 69.68	152.55 ± 70.60	0.006	0.938
N3 stage duration [min]	63.88 ± 66.29	64.16 ± 62.70	0.025	0.816
REM stage duration [min]	74.60 ± 58.49	75.09 ± 62.21	0.048	0.431
Sleep latency, min	15.85 ± 21.66	11.26 ± 12.24	1.538	0.578
REM latency, min	102.71 ± 66.25	102.85 ± 75.72	−0.011	0.126
Total AHI [events/h]	21.15 ± 10.22	20.40 ± 10.54	0.428	0.669
AHI REM, events/h	18.04 ± 11.63	14.42 ± 8.29	2.111	0.037
NREM-AHI [events/h]	6.49 ± 13.16	6.41 ± 7.51	0.047	0.963
ODI, events/h	20.41 ± 14.72	15.42 ± 12.95	2.128	0.035
Longest apnea time, s	31.55 ± 11.23	31.55 ± 11.23	2.520	0.013
Longest hypoventilation time, s	101.27 ± 21.51	90.24 ± 28.75	2.574	0.011
Min SpO_2_ [%]	84.39 ± 5.19	86.58 ± 6.23	−2.258	0.025
Respiratory event-related	25.04 ± 14.33	25.64 ± 15.09	−0.240	0.811
Arousal index, events/h	8.22 ± 11.36	5.04 ± 4.69	2.157	0.033

WASO, wake time after sleep onset; REM, rapid eye movement; AHI, apnea hypopnea index; ODI, oxygen desaturation index.

### 3.4 Differences in neurological function, prognosis, and sleep-related scores

Neurological function, prognosis, and sleep-related scale scores are presented in Table 4. The results showed statistically significant differences between the two groups in follow-up NIHSS scores, 3-month mRS scores, and ESS scores. No significant differences were observed between groups with respect to pre-follow-up NIHSS scores, pre-follow-up mRS scores, BI, PSQI scores, HADS-A scores, or HADS-D scores. The PSQI was used as a supplementary instrument to assess overall subjective sleep quality and to describe patients’ perceived sleep status; no significant sex-related differences were observed in PSQI scores.

**Table 4:** Comparison of gender differences in neurological function, prognosis, and sleep-related scores among patients in the two groups.

Variables	Female (*N* = 71)	Male (*N* = 69)	*t*	*p*
NIHSS (before)	6.20 ± 1.97	6.13 ± 1.77	0.211	0.833
NIHSS (after)	4.17 ± 1.63	3.49 ± 1.45	2.590	0.011
MRS (before)	3.07 ± 0.96	3.09 ± 1.29	−0.086	0.932
MRS (after)	1.27 ± 0.74	0.93 ± 0.75	2.701	0.008
Barthel score	47.44 ± 6.77	48.13 ± 7.15	−0.590	0.556
ESS score	7.04 ± 4.98	5.29 ± 3.95	2.303	0.023
PSQI score	18.55 ± 6.37	20.35 ± 4.48	−1.928	0.056
HADSA score	2.31 ± 2.77	2.42 ± 2.38	−0.253	0.801
HADSD score	2.00 ± 2.79	2.01 ± 2.50	−0.032	0.974

NIHSS, National Institutes of Health Stroke Scale; mRS, Modified Rankin Scale; Barthel, Barthel Index of Activities of Daily Living; ESS, Epworth Sleepiness Scale; PSQI, Pittsburgh Sleep Quality Index; HADS, Hospital Anxiety and Depression Scale.

**Figure 2:**
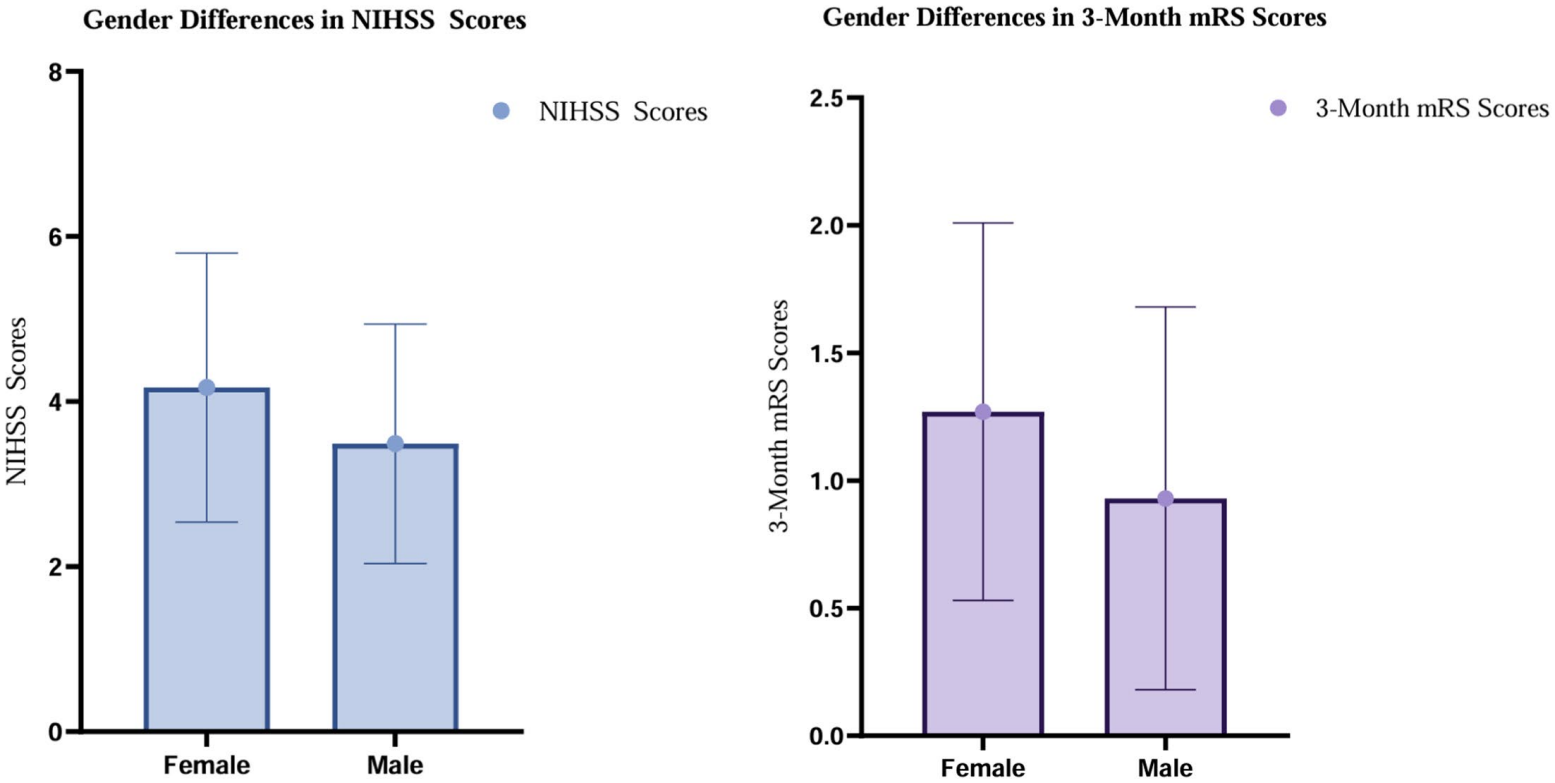
Gender differences in NHISS and 3-month mRS scores.

Overall, these findings suggest an unfavorable trend in neurological outcomes and disability severity during follow-up among female patients. It should be noted that although ESS scores differed significantly between sexes, the absolute ESS values in both groups remained within the normal range, indicating no clinically meaningful excessive daytime sleepiness (see Figure 2).

### 3.5 Results of influencing factor analysis

To identify factors associated with 3-month mRS scores in female patients, a multivariable linear regression analysis was performed incorporating variables including smoking status, white blood cell count, neutrophil count, total sleep time, and respiratory event–related parameters. The results are presented in Table 5.

**Table 5:** Results of multiple linear regression analysis for 3-month post-follow-up mRS score.

Variables	*B*	SE	*β*	*t*	*p*	95%(CI)
Smoking history [*n*, %]	−0.134	0.210	−0.071	−0.638	0.526	(−0.554, 0.286)
WBC, 10^9^/L	0.027	0.024	0.121	1.133	0.262	(−0.021, 0.076)
NE, 10^9^/L	0.018	0.007	0.258	2.404	0.019	(0.003, 0.033)
ESS score	0.002	0.015	0.015	0.144	0.886	(−0.029, 0.033)
Total sleep time, min	−0.001	0.001	−0.055	−0.488	0.637	(−0.003, 0.002)
Sleep efficiency	0.007	0.008	0.093	0.843	0.403	(−0.010, 0.024)
Sleep latency, min	−0.006	0.004	−0.191	−1.797	0.078	(−0.014, 0.001)
AHI REM, events/h	0.018	0.007	0.285	2.625	0.011	(0.004, 0.032)
ODI, events/h	0.005	0.005	0.095	0.889	0.378	(−0.006, 0.015)
Longest hypoventilation time, s	0.005	0.003	0.139	1.362	0.178	(−0.002, 0.012)
Longest apnea time, s	−0.002	0.007	−0.024	−0.241	0.810	(−0.015, 0.012)
Min SpO₂ [%]	−0.046	0.016	−0.325	−2.872	0.006	(−0.078, −0.014)
Arousal index, events/h	0.004	0.007	0.062	0.595	0.554	(−0.010, 0.018)

*F* = 3.819, *p* < 0.001, *R*^2^ = 0.344, adjusted *R*^2^ = 0.344.

During model construction, both global indices reflecting the overall severity of sleep-disordered breathing and sleep stage–specific respiratory parameters were simultaneously included to comprehensively assess the impact of total disease burden and stage-dependent respiratory events on prognosis.

The analysis demonstrated that neutrophil count was positively associated with mRS scores, indicating that higher neutrophil levels were related to worse functional outcomes at 3 months in female patients. REM-AHI was also positively correlated with mRS scores, suggesting that more frequent REM-related apnea–hypopnea events were associated with greater disability. In contrast, the lower nadir of oxyhemoglobin saturation was negatively correlated with mRS scores, indicating that lower minimal oxygen saturation was associated with poorer functional outcomes. Smoking status, white blood cell count, ESS score, total sleep time, sleep efficiency, sleep latency, ODI, longest hypopnea duration, longest apnea duration, and respiratory-related arousal index were not significantly associated with 3-month mRS scores in female patients (all *p* > 0.05).

**Figure 3:**
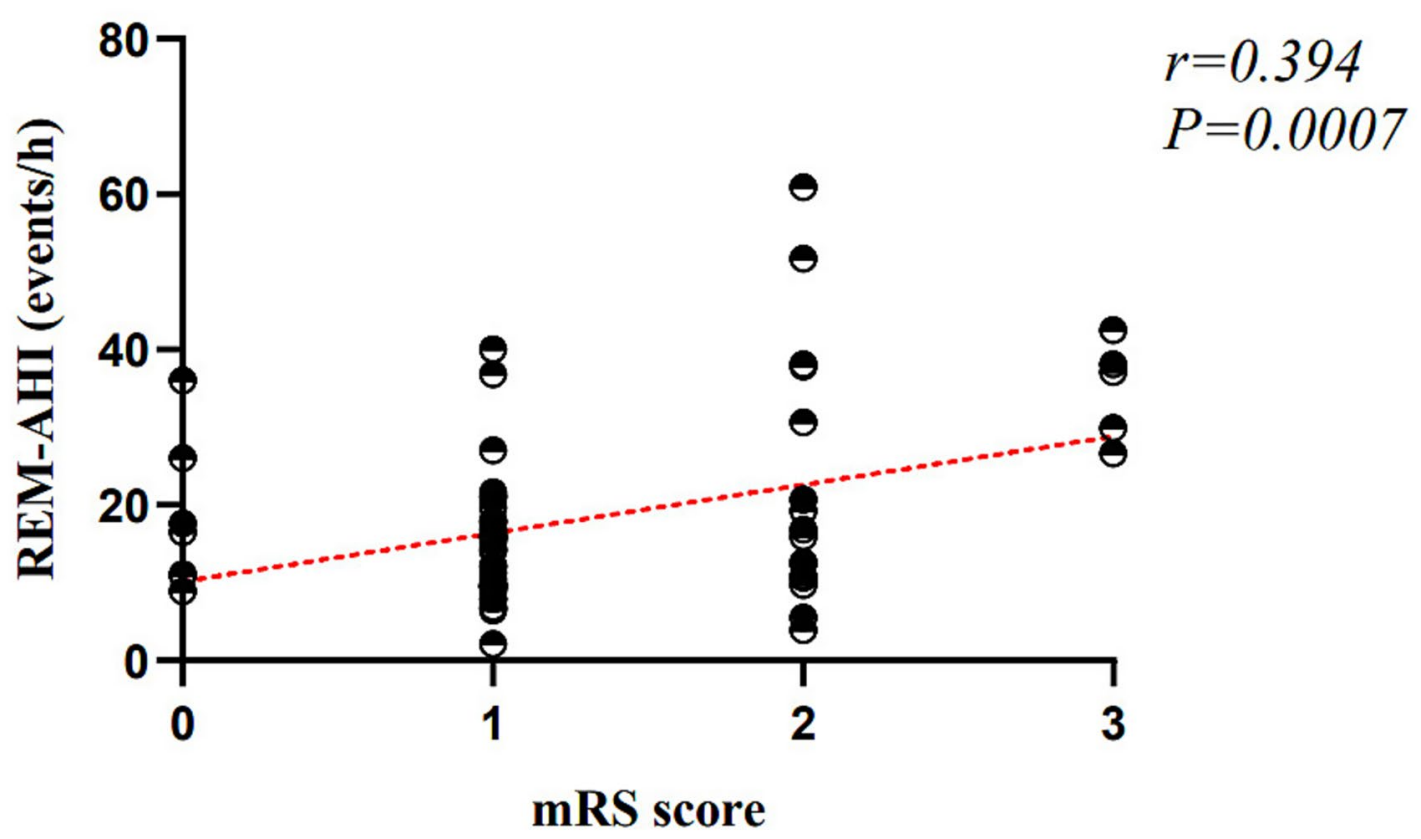
Association between mRS score and REM-AHI in female patients.

**Figure 4:**
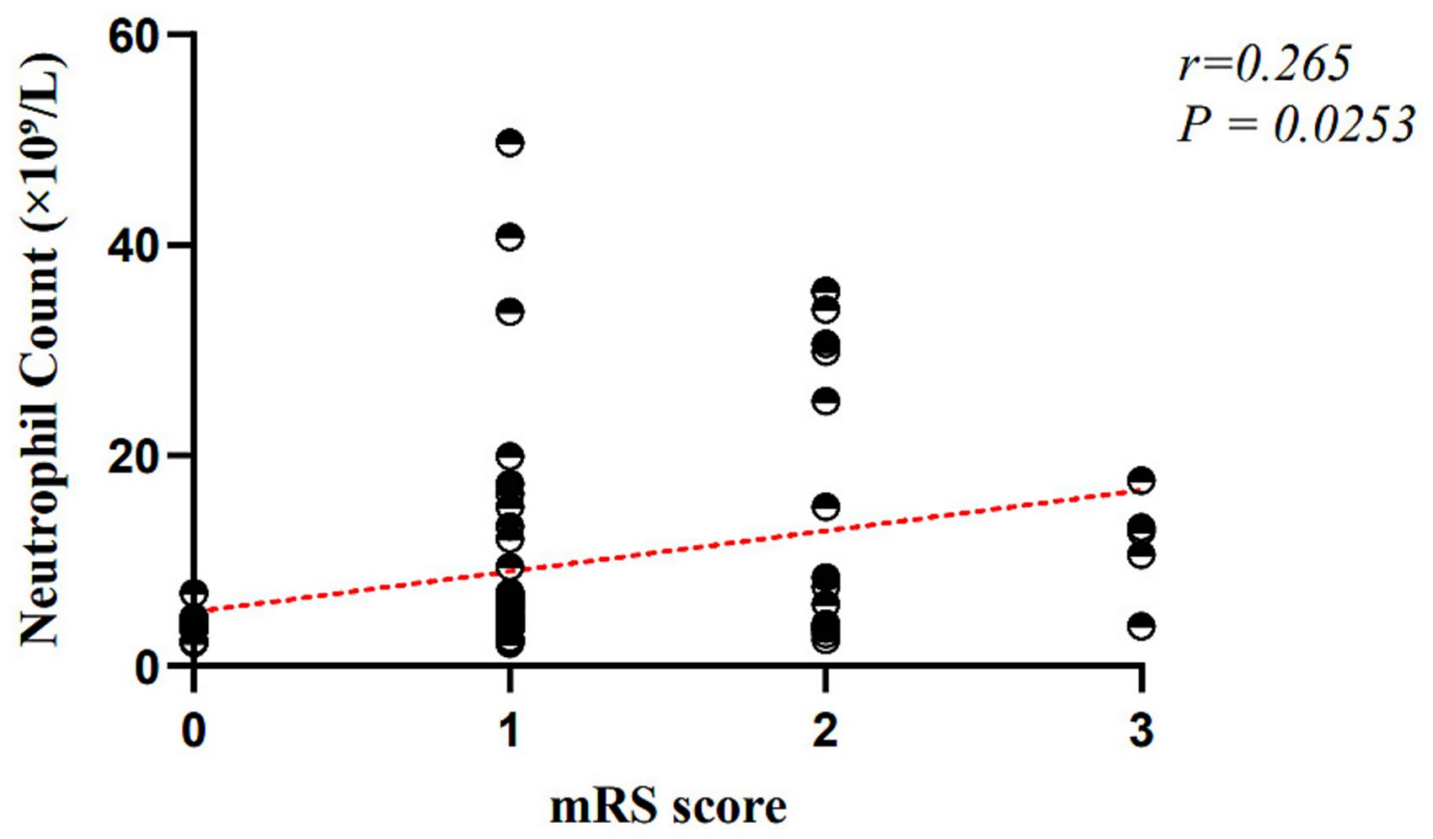
Simple linear regression of neutrophil count and mRS score in female patients.

**Figure 5:**
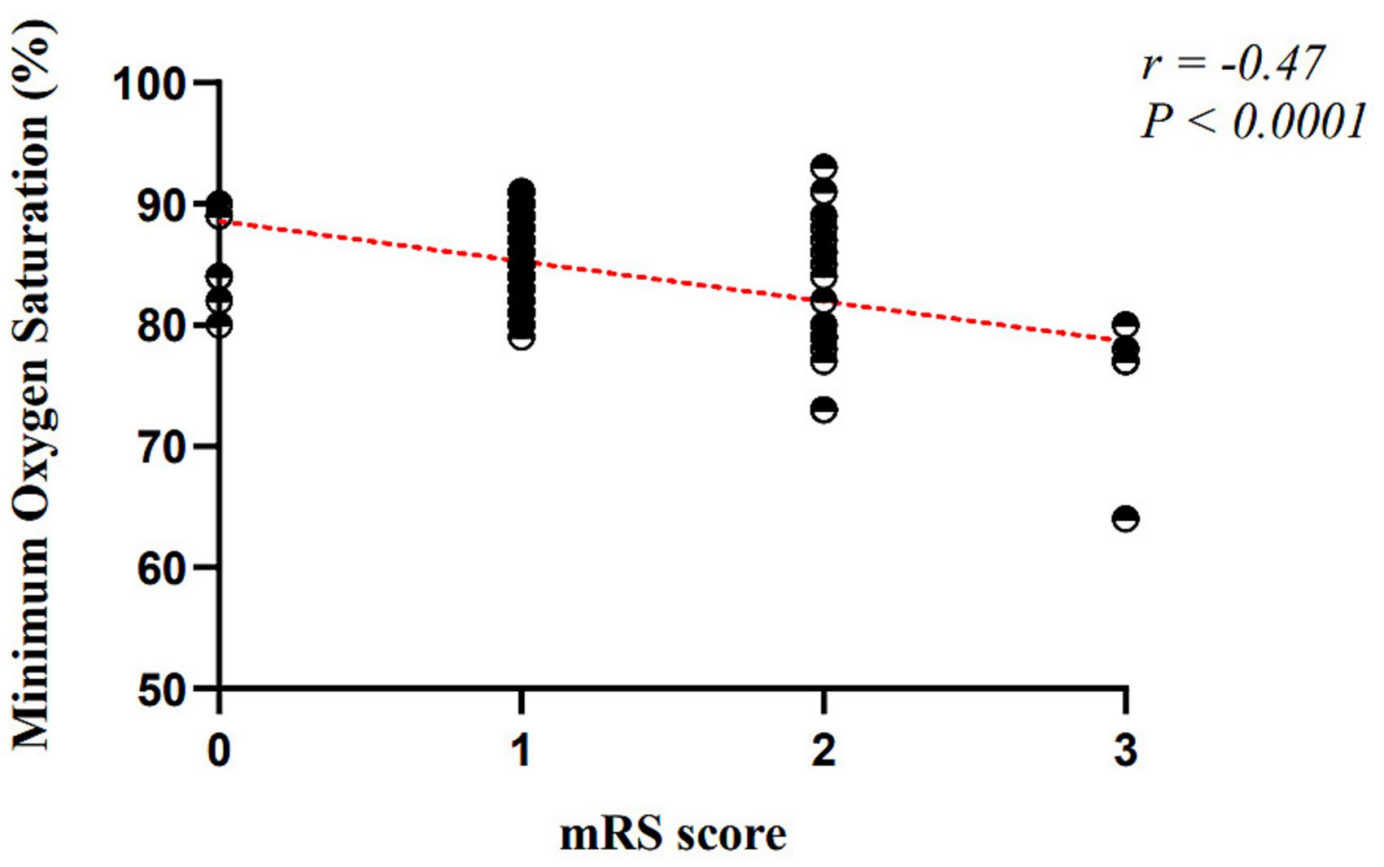
Linear regression of minimum oxygen saturation and mRS score in female patients.

The overall model was statistically significant (*F* = 3.819, *p* < 0.001), with an adjusted *R*^2^ of 0.344, indicating good explanatory power (see Figures 3–5).

## 4 Discussion

This study focused on gender differences between male and female patients with ACI complicated by REM-OSA, aiming to clarify the impact of gender on disease characteristics and prognosis. The study found that female patients with ACI complicated by REM-OSA had worse prognosis; these female patients had significantly higher white blood cell count and neutrophil count, and more disorganized sleep structure. The possible reason was that REM-AHI, minimum blood oxygen saturation, and neutrophil count were independent predictors of prognosis in female patients with ACI complicated by REM-OSA. Among these factors, REM-AHI served as a core predictor, and its pathological significance stems from the unique physiological characteristics of the REM sleep stage—which is accompanied by highly active neuronal activity, reduced skeletal muscle tone, and dream-related neuroelectric activity (19), and exhibits significantly higher sensitivity to hypoxia and respiratory disturbances than other sleep stages (20). Elevated REM-AHI induces REM stage-specific abnormal respiratory rhythm, which directly leads to a decrease in Nadir SpO₂ (minimum blood oxygen saturation) and forms a “respiratory disturbance-hypoxic injury” cascade effect. This causal relationship renders it a key indicator for prognostic assessment. For patients with ACI, REM stage respiratory abnormalities can amplify ischemic brain injury through multiple pathological mechanisms; its association with prognosis exhibits higher specificity and clinical guiding value, thus it is emphasized herein. The suppression of upper airway muscle group activation during the REM sleep stage is significantly stronger than that during the NREM sleep stage. Studies have confirmed that compared with NREM sleep, REM-stage obstructive apnea has a longer duration, more severe hypoxemia, and a greater decrease in apnea-related blood oxygen desaturation (21). This phenomenon is closely associated with reduced upper airway muscle tone during REM sleep (22, 23), decreased reflex sensitivity of the genioglossus muscle to negative pressure stimulation (24, 25), and weakened chemoreceptor regulatory function (26, 27), which collectively exacerbate the severity of OSA during REM sleep (28). Abnormal elevation of REM-AHI can directly lead to a significant decrease in nocturnal Nadir SpO₂ in female patients, while triggering a more intense inflammatory response and oxidative stress, thereby promoting the excessive release of proinflammatory cytokines (29). Existing studies have confirmed that vascular endothelial dysfunction and inflammatory activation are key driving mechanisms of OSA-related vascular lesions (30). However, the REM stage-specific physiological characteristics further exacerbate the secondary damage of ischemic brain tissue by affecting cerebral hemodynamics and aggravating vascular endothelial injury; thus, REM-AHI exhibits particularly prominent prognostic predictive value. As a direct pathological outcome of abnormal REM-AHI elevation, Nadir SpO₂ serves as another important independent predictor for prognostic assessment in this population. The present study showed that Nadir SpO₂ in female ACI patients was significantly lower than that in male patients, and was closely associated with poor prognosis; this difference is mainly attributed to persistent hypoxic exposure caused by elevated REM-AHI in females. As an objective indicator of impaired oxygenation function in the body (31), decreased blood oxygen saturation induces oxygen imbalance, which leads to hypoxia, hyperoxia, protein misfolding, mitochondrial dysfunction, altered heme metabolism, and neuroinflammation (32). Furthermore, long-term intermittent hypoxia may result in persistent neuroinflammation, which is a key driving factor for neurodegenerative diseases (33). The significant correlation between the frequency of respiratory events and the magnitude of blood oxygen saturation decrease in REM-OSA patients further confirms the causal relationship between the two and their synergistic impact on prognosis.

Given the concealment of REM-OSA-related nocturnal hypoxia, clinical practice requires strengthening the combined assessment of REM-AHI and Nadir SpO₂ in PSG monitoring to accurately identify high-risk populations.

Among the three aforementioned independent predictors, the mechanism underlying neutrophil count also deserves in-depth discussion. Neutrophilia, a pathological state characterized by abnormally elevated numbers of neutrophils in peripheral blood, is commonly observed in various pathological processes such as infection and inflammatory diseases (34). It damages vascular endothelial cells by releasing a variety of inflammatory mediators and promotes venous thrombosis (especially acute thrombosis) (35). In ACI patients, neutrophil levels are positively correlated with infarct volume and the degree of neurological deficit, suggesting that the inflammatory cascade mediated by neutrophils may directly participate in the pathophysiological process of ACI through pathways such as promoting thrombosis and exacerbating microcirculatory disturbance.

The results of this study showed that the WBC count and NE count in female patients with ACI complicated by REM-OSA were significantly higher than those in male patients. This difference may be related to multiple factors. From a physiological perspective, female hormone levels undergo significant changes during special periods such as the menstrual cycle, pregnancy, and menopause (36). Studies have suggested that estrogen can regulate the activity and proliferation of immune cells to a certain extent, which may promote the increase in the production of WBCs and neutrophils (37). In addition, menopause exerts a significant impact on the immune system in women; postmenopausal women are more susceptible to infections, which may also lead to elevated WBC and NE counts (38). In terms of pathology, recurrent apnea and hypopnea at night in REM-OSA patients result in chronic intermittent hypoxia (CIH) in the body (3). This state of CIH can induce a series of inflammatory responses in the body, such as inducing the expression and secretion of inflammation-related cytokines (39). The inflammatory mediators released by these cells further aggravate vascular endothelial injury and promote thrombosis, thereby exacerbating brain tissue damage in patients with acute cerebral infarction (30).

To summarize, REM-AHI, Nadir SpO₂, and neutrophil count are all associated with the prognosis of female patients with ACI complicated by REM-OSA. Among these factors, REM-AHI plays a more critical role in prognostic assessment due to the unique physiological mechanisms of the REM sleep stage, and thus should be prioritized for attention and intervention in clinical practice.

From the perspective of specific sleep characteristics: In terms of sleep data, this study showed that female patients exhibited more severe sleep architecture disruption, more serious REM-stage apnea, and worse oxygenation status. At the physiological structure level, female patients differ from male patients in upper airway anatomical characteristics (40); their airway lumen area from the 4th to 6th generations is smaller, leading to increased airway resistance (41). This anatomical feature makes females more prone to upper airway collapse during the REM stage, thereby inducing apnea and hypopnea events. In terms of clinical characteristics, atypical symptoms of sleep apnea are more common in females, and the distribution of apnea events during the REM stage is significantly higher than that during the NREM stage (42). Furthermore, hormonal fluctuations exert a significant impact on female sleep; women with irregular menstrual cycles report more complaints of sleep difficulties, which further exacerbates sleep architecture disruption (43). The aforementioned sleep abnormalities induce nocturnal hypoxia through recurrent apnea and hypopnea. Notably, OSA-related hypoxic stress can affect cardiac function by inducing arrhythmias and conduction disorders (44), while long-term sleep architecture disruption can involve the cardiovascular system and lead to cognitive impairment (memory, executive function) as well as brain atrophy (45).

In terms of gender differences in prognostic outcomes, studies have shown that while there were no gender differences in baseline neurological deficit (NIHSS), disability level (mRS), and sleep disorder scores (PSQI, HADS) at admission, female patients exhibited significantly higher NIHSS and mRS scores at the 3-month follow-up. This suggests that females may have specific pathophysiological repair mechanisms and sociobiological risk profiles, leading to delayed neurological function recovery and exacerbated long-term disability; the underlying mechanism may be associated with sleep and inflammatory indicators. At the sleep level, females have poorer sleep quality and higher REM-AHI (46), which directly prolongs the duration of nocturnal hypoxia and results in insufficient cerebral oxygen supply. Hypoxia can exacerbate the damage of cerebral infarction foci and impede neurological function recovery (2); further studies have confirmed that nocturnal hypoxia can induce degeneration of cortical and brainstem neurons as well as axonal dysfunction, thereby affecting brain function (47). Meanwhile, OSA-related hypoxia promotes the release of proinflammatory cytokines through neutrophil-vascular endothelial cell interactions, exacerbating inflammation and oxidative stress (48). Under stress, vasoactive substances secreted by vascular endothelial cells can cause abnormal vasodilation and microcirculatory disturbance, transforming their phenotype from anti-adhesive and anti-coagulant to pro-adhesive and pro-coagulant, which accelerates thrombosis and aggravates cerebral infarction (49). At the treatment level, females exhibit gender-specific differences in the tolerance and responsiveness to certain drugs (e.g., paracetamol, metoprolol), with a significantly higher risk of adverse drug reactions (approximately twice that of males) (50, 51), which may further affect prognosis.

In conclusion, this study confirms the presence of significant gender differences in ACI patients complicated with REM-OSA, and identifies REM-AHI as a core prognostic indicator for females. This provides important evidence for formulating gender-specific intervention strategies.

## 5 Implications of the study findings for clinical treatment

The findings of this study provide important guidance for clinical diagnosis and treatment. It is recommended that clinical practice strengthen the screening of REM-OSA in female patients with ACI. Given that females present with atypical symptoms which make them prone to misdiagnosis, it is advisable to routinely perform sleep respiratory monitoring in female ACI patients to achieve early identification. Treatment should implement individualized regimens based on gender differences. For female patients, while actively managing ACI and REM-OSA, focus should be placed on inflammatory responses and hormonal changes: for those with significant inflammation, anti-inflammatory therapy may be administered as appropriate to alleviate brain and vascular damage; for those with abnormal hormone levels (e.g., postmenopausal patients), hormone replacement therapy may be considered after a risk–benefit assessment. During the rehabilitation phase, it is necessary to enhance the management and guidance of female patients, improve treatment adherence through regular follow-up and health education to optimize rehabilitation outcomes, and meanwhile attach importance to mental health. Timely intervention for emotional issues such as anxiety and depression should be conducted to promote comprehensive recovery.

## 6 Unique findings and significance of the study

This study has made innovative findings regarding gender differences and prognostic factors in patients with ACI complicated by REM-OSA. In terms of disease association, it confirms that REM-OSA can significantly exacerbate the condition of ACI and affect prognosis, which is consistent with the conclusions of existing studies. Regarding gender differences: although the overall incidence of OSA is higher in males, in the specific population of ACI patients complicated with REM-OSA, female patients show significant differences in the severity of sleep architecture disruption, the severity of REM-stage apnea, and clinical prognosis. This is attributed to the study’s strict definition of research subjects and the inclusion of comprehensive sleep monitoring data and laboratory indicators for in-depth analysis. In terms of prognostic factors: in addition to verifying the importance of traditional parameters such as peripheral blood neutrophils and blood oxygen saturation, the study clearly identifies the AHI as an independent risk factor affecting the prognosis of female ACI patients complicated with REM-OSA. This achievement benefits from the targeted selection of research subjects and the use of multiple linear regression analysis for systematic screening of potential prognostic factors.

In summary, this study has deepened the understanding of the pathophysiological characteristics and prognostic mechanisms of ACI patients complicated with REM-OSA, particularly revealing the value of gender-specific differences and novel biological markers. It provides an important theoretical basis and targeted references for the implementation of more precise individualized diagnosis and treatment strategies in clinical practice.

## 7 Limitations of the study

This study has several limitations that should be acknowledged. First, the relatively small sample size (*n* = 140) may have limited the representativeness and generalizability of the findings, making it difficult to fully capture the overall characteristics of the target population. Second, the baseline National Institutes of Health Stroke Scale (NIHSS) scores of the included patients were predominantly within the mild-to-moderate range, indicating that the study population mainly consisted of patients with mild-to-moderate acute ischemic stroke (ACI). This may have introduced selection bias and limited the extrapolation of the results to patients with severe stroke.

In addition, ischemic stroke subtypes were not further stratified in the present study. Given the marked heterogeneity of acute ischemic stroke, different stroke subtypes may vary in etiological mechanisms, risk factor distributions, stroke severity, and clinical outcomes, which could influence the interpretation and generalizability of the results (52). Previous studies have also demonstrated that stroke subtype is an important determinant of clinical outcomes (53). Moreover, the specific distribution of ischemic stroke subtypes within the study population was not separately described or compared, which may have further limited a more in-depth interpretation of the findings.

Furthermore, the anatomical location of ischemic stroke lesions was not systematically analyzed. Prior studies have suggested that involvement of specific brain regions (such as the internal capsule or pons), particularly among smokers, is associated with a higher prevalence or greater severity of sleep-related breathing disorders (54). Therefore, stroke topography may represent a potential confounding factor influencing both sleep-disordered breathing characteristics and prognosis. The lack of adequate control for this factor may have constrained the interpretation and external validity of the results.

Additionally, as all participants were recruited from a single center, the study is subject to regional limitations, which may reduce the applicability of the findings to other populations or geographic settings. Sleep assessment was based on a single overnight polysomnography, which may be influenced by night-to-night variability and may not comprehensively reflect the long-term characteristics of sleep-disordered breathing.

Finally, due to the retrospective design, the study is inherently susceptible to selection bias, information bias, and uncontrolled confounding during data collection, which may have affected the accuracy of the results. Future studies should aim to include larger sample sizes, adopt multicenter collaborative designs, increase the frequency of sleep monitoring or employ longitudinal monitoring approaches, and utilize prospective study designs to further validate and refine the conclusions of the present study.

## 8 Conclusions and prospects

### 8.1 Summary of the main conclusions of the study

This study found that among patients with ACI complicated by REM-OSA, females have a significantly worse prognosis than males. REM-AHI, Nadir SpO₂, and neutrophil count are key factors affecting females’ prognosis, suggesting that sleep-disordered breathing, hypoxia, and inflammatory response are the core mechanisms underlying females’ poor prognosis. Therefore, in clinical practice, priority should be given to attention to female patients to optimize prognostic assessment and intervention.

### 8.2 Outlook on future research directions

Based on the limitations and findings of this study, future research can focus on the following aspects: conducting large-sample, multi-center, and cross-ethnic cohort studies to enhance the extrapolatability of conclusions; carrying out in-depth analysis of the molecular mechanisms of the sex hormone-inflammation-sleep breathing regulatory axis to provide targets for precision typing; advancing randomized controlled trials targeting female-specific pathological phenotypes to verify the efficacy of targeted interventions; and establishing a long-term prospective prognostic monitoring system to quantify long-term risks and construct a risk stratification model.

## Data Availability

The original contributions presented in the study are included in the article/supplementary material, further inquiries can be directed to the corresponding authors.
